# Challenges and opportunities in neonatal monitoring: insights on forehead photoplethysmography sensors

**DOI:** 10.1038/s41390-025-04376-4

**Published:** 2025-09-22

**Authors:** Lena Strasser, Tereza Schedler, Robyn Dvorsky, Michael Wagner

**Affiliations:** Division of Neonatology, Pediatric Intensive Care and Neuropediatrics, Department of Pediatrics, Comprehensive Center for Pediatrics, Medical University of Vienna, Vienna, Austria

During the transition after birth, an infant’s heart rate (HR) is the most important determinant used to decide whether further resuscitative interventions are necessary. It is considered the most sensitive indicator of the efficacy of resuscitation.^1,2^ Furthermore, preductal oxygen saturation (SpO_2_) also plays an important role in the decision-making process for providing oxygen and respiratory support. Therefore, continuous monitoring of these two parameters during stabilization and resuscitation of newborns at risk or with poor adaptation is recommended by the European Resuscitation Council.^1^ However, achieving accurate HR and SpO_2_ measurements during the transition from fetal to newborn life can be challenging and is still a highly controversial scientific topic. For instance, a recently published systematic review showed that pulse oximetry is less accurate than electrocardiography (ECG) in the determination of HR after birth.^2^ Based on the evidence provided in previous trials, ECG is recommended for monitoring HR after birth.^2^ Strictly speaking, however, ECG does not provide direct information about cardiac output or mechanical function, but rather records the heart’s electrical activity. Furthermore, ECG electrode placement can be difficult right after birth, which can cause issues with signal pick-up. Moreover, it remains unclear if using ECG or pulse oximetry for HR monitoring has an effect on patient outcomes.^3^

Promising new technologies are on the rise but require further investigation before implementation in standard clinical care and guidelines. For instance, photoplethysmography (PPG) is a method for oxygen saturation monitoring apart from peripheral pulse oximetry. Both technologies utilize optical methods to measure physiological parameters but differ in their application and adaptability. PPG detects changes in blood volume using light reflection or transmission and can be applied to various body sites, such as the forehead, making it less dependent on peripheral perfusion. In contrast, peripheral pulse oximetry relies on light transmission through thin, well-perfused peripheral tissues such as the fingers or earlobes, making it susceptible to motion artifacts and reduced signal quality under conditions of poor perfusion.^4,5^

Addressing these pending issues, Swamy et al. conducted a clinical trial comparing two vital sign sensors placed either at the wrist or at the forehead during the transition after birth. Within this study, 20 healthy term newborns, delivered via elective cesarian section, were monitored with a wireless forehead PPG sensor (SurePulse Medical, Nottingham, United Kingdom), an ECG (SKINTACT, Leonhard Lang GmbH, Innsbruck, Austria) and a wrist pulse oximetry sensor (LNCS Neo, Masimo, Irvine, California, USA), in this particular order. This clinical trial demonstrated the feasibility of vital parameter monitoring during neonatal transition using a forehead sensor. Their findings include accurate HR measurement of both sensors compared to ECG, but significantly higher SpO_2_ measurements from the forehead sensor compared to the wrist sensor. Furthermore, the “success heart rate” of the forehead sensor, which is described as the percentage of possible HR measurements after sensor placement, was also significantly higher than the success HR of the wrist sensor.^6^ The accuracy of HR monitoring in NICU patients and newborns after cesarian section using the forehead sensor was previously studied by the same research group. Their findings speak in favor of accurate and reliable HR measurements provided by the wireless PPG forehead sensor.^7^

Arterial oxygen saturation is a fundamental parameter that provides accurate information about oxygenation, whereas peripheral SpO_2_ serves as a surrogate marker, that can be influenced by a variety of other factors.^8^ Regarding the observed differences in measured oxygen saturation, Swamy et al. emphasize that their study does not determine which sensor’s measurements correspond closest to arterial oxygen saturation.^6^ Referring to this, it should be kept in mind that pulse oximetry seems to be insufficient for the determination of arterial oxygen saturation and tension in newborns.^8^ The observed differences in SpO_2_ measurements from the forehead compared to the wrist sensor are in line with previous literature, as other trials also found differences in pulse oximeter readings from different pulse oximeter types. For instance, Maiwald et al. recently performed a crossover trial aiming to compare pulse oximetry obtained from three different sensor types produced by Masimo, Irvine, California, USA (Red Diamond, Low Noise Cabled Sensor (LNCS), Radius PPG sensor) in extremely preterm neonates. They found significant differences of readings from different sensors, with higher SpO_2_ readings from the wireless PPG sensor and lower SpO_2_ readings from the Red Diamond sensor compared to LNCS, whereas the difference between Red Diamond and LCNS was larger.^9^ Swamy et al. suggest that regarding their trial, higher perfusion of the forehead and brain through the carotid arteries to the disadvantage of peripheral regions may have resulted in higher oxygen saturation levels measured via the PPG sensor. Additionally, they state that the forehead and wrist differ vastly in terms of tissue composition.^6^ Further investigation is encouraged to develop procedures and devices that measure arterial saturation as accurately as possible. Moreover, it is crucial to develop strategies to adapt to the differences in oxygen saturation measurements from different sensors, such as redefined target saturation levels, depending on the respective measurement tool. Thereby, oxygen or respiratory support could be used in an even more targeted manner and, thus, ultimately patient outcomes might be improved by avoiding both hypoxia and hyperoxia as suggested by the ERC.^1^

In this context, the user friendliness of medical devices should also be a key component in clinical studies. It would be interesting to investigate whether the placement of a single sensor for monitoring influences provider stress and the time required for monitoring as well as the time required for obtaining valid vital parameter measurements. One key component of the PPG sensor is its wireless design, as the use of wireless monitoring devices is of growing scientific interest in neonatology and pediatrics and may be beneficial for clinicians as wires can lead to sensor detachment and difficulties while providing patient care, especially when repositioning the patient. However, literature suggests that there is still a need for high quality research to ensure safe and appropriate clinical use.^10^ Notwithstanding potential benefits of a wireless forehead sensor, it should still be considered, that during the trial, five patients had to be excluded because removal of the forehead sensor was necessary for resuscitative measures or other interventions not further described in the original article.^6^ An important aspect in the feasibility of vital parameter monitoring with a forehead sensor is its application during newborn life support, especially the compatibility or simultaneous use with respiratory devices such as continuous positive airway pressure masks, which should be explored in future trials.

Finally, the broader clinical implications of using forehead sensors, such as their impact on clinical decision-making, health care provider stress and neonatal outcomes, should be investigated. Evaluating whether the faster and more reliable measurement of HR and SpO_2_ using forehead sensors could improve response times or reduce intervention rates, would provide valuable insights into their potential role within neonatal care. Despite the limitations of the trial, which are described in the original article, such as a relatively low sample only consisting of healthy term newborns, these results suggest the potential of forehead PPG sensors to provide a deeper insight into the transition from fetal to neonatal life. Investigating this transition could bring us one step closer to providing high-quality and effective newborn care and support.
